# Modulation of Arterial Stiffness Gradient by Acute Administration of Nitroglycerin

**DOI:** 10.3389/fphys.2021.774056

**Published:** 2021-12-15

**Authors:** Catherine Fortier, Charles-Antoine Garneau, Mathilde Paré, Hasan Obeid, Nadège Côté, Karine Duval, Rémi Goupil, Mohsen Agharazii

**Affiliations:** ^1^CHU de Québec Research Center-Université Laval, L’Hôtel-Dieu de Québec Hospital, Québec, QC, Canada; ^2^Division of Nephrology, Faculty of Medicine, Université Laval, Québec, QC, Canada; ^3^Research Center of the Hôpital du Sacré-Coeur de Montréal, Montréal, QC, Canada

**Keywords:** arterial stiffness gradient, arterial stiffness, aortic stiffness, pulse wave velocity (PWV), nitroglycerin (NTG), pulse wave analysis (PWA), chronic kidney disease (CKD), arterial compliance

## Abstract

**Background:** Physiologically, the aorta is less stiff than peripheral conductive arteries, creating an arterial stiffness gradient, protecting microcirculation from high pulsatile pressure. However, the pharmacological manipulation of arterial stiffness gradient has not been thoroughly investigated. We hypothesized that acute administration of nitroglycerin (NTG) may alter the arterial stiffness gradient through a more significant effect on the regional stiffness of medium-sized muscular arteries, as measured by pulse wave velocity (PWV). The aim of this study was to examine the differential impact of NTG on regional stiffness, and arterial stiffness gradient as measured by the aortic-brachial PWV ratio (AB-PWV ratio) and aortic-femoral PWV ratio (AF-PWV ratio).

**Methods:** In 93 subjects (age: 61 years, men: 67%, chronic kidney disease [CKD]: 41%), aortic, brachial, and femoral stiffnesses were determined by cf-PWV, carotid-radial (cr-PWV), and femoral-dorsalis pedis artery (fp-PWV) PWVs, respectively. The measurements were repeated 5 min after the sublingual administration of NTG (0.4 mg). The AB-PWV and AF-PWV ratios were obtained by dividing cf-PWV by cr-PWV or fp-PWV, respectively. The central pulse wave profile was determined by radial artery tonometry through the generalized transfer function.

**Results:** At baseline, cf-PWV, cr-PWV, and fp-PWV were 12.12 ± 3.36, 9.51 ± 1.81, and 9.71 ± 1.89 m/s, respectively. After the administration of NTG, there was a significant reduction in cr-PWV of 0.86 ± 1.27 m/s (*p* < 0.001) and fp-PWV of 1.12 ± 1.74 m/s (*p* < 0.001), without any significant changes in cf-PWV (*p* = 0.928), leading to a significant increase in the AB-PWV ratio (1.30 ± 0.39 vs. 1.42 ± 0.46; *p* = 0.001) and AF-PWV ratio (1.38 ± 0.47 vs. 1.56 ± 0.53; *p* = 0.001). There was a significant correlation between changes in the AF-PWV ratio and changes in the timing of wave reflection (*r* = 0.289; *p* = 0.042) and the amplitude of the heart rate-adjusted augmented pressure (*r* = − 0.467; *p* < 0.001).

**Conclusion:** This study shows that acute administration of NTG reduces PWV of muscular arteries (brachial and femoral) without modifying aortic PWV. This results in an unfavorable profile of AB-PWV and AF-PWV ratios, which could lead to higher pulse pressure transmission into the microcirculation.

## Introduction

Aortic stiffness, determined by aortic pulse wave velocity (PWV), is a hallmark of vascular aging and an independent cardiovascular risk factor in various clinical conditions ranging from hypertension to end-stage kidney disease (Blacher et al., 1999a,b; Laurent et al., 2001; Cruickshank et al., 2002; Karras et al., 2012). The consequences of aortic stiffening on the heart are essentially explained by an increased cardiac workload and a decreased coronary perfusion pressure. However, the peripheral target organ damage may best be explained by the arterial stiffness gradient hypothesis (Mitchell, 2008). Indeed, due to the heterogeneity of vascular wall composition and diameter, there is an increase in arterial stiffness (stiffness gradient) from the heart to the periphery. This gradient in arterial stiffness results in a gradual attenuation of forward pressure wave along the arterial tree down to the microcirculation, where the pulsatility must be minimal. In the process of vascular aging, medium-sized arteries show little changes compared to the aorta, leading to attenuation or even a reversal of the stiffness gradient (Mitchell, 2008; Fortier and Agharazii, 2016). As a consequence, a higher pulse pressure could be transmitted into the microcirculation, causing exaggerated vascular myogenic response, and ultimately hypoperfusion and organ damage (Mitchell, 2008; Fortier and Agharazii, 2016). Indeed, a number of recent studies have underlined the relevance of arterial stiffness gradient on both blood flow and clinical outcomes (Mitchell et al., 2011; Tarumi et al., 2011; Hashimoto and Ito, 2013).

In the context of chronic kidney disease (CKD), we have previously used the PWV ratio between cf-PWV and cr-PWV as a surrogate measure of arterial stiffness gradient and have shown that it was a better prognostic indicator than cf-PWV alone (Fortier et al., 2015). In CKD patients, we have shown that the changes in arterial stiffness gradient are not only guided by an increase in aortic stiffness but also by a decrease in the stiffness of the medium-sized muscular arteries such as the brachial artery (Utescu et al., 2013; Fortier et al., 2015). We have also observed that patients with chronic use of nitroglycerin (NTG), a vasodilatory drug used primarily to treat coronary artery disease and/or heart failure, had a higher PWV ratio. This observation led us to hypothesize that NTG may have a negative impact on arterial stiffness gradient given that it has a more pronounced effect on medium-sized conduit arteries where vascular smooth muscle cells are a major component of the arterial wall (Wilkinson et al., 2002; Pauca et al., 2005; Yamamoto et al., 2017). However, the pharmacological manipulation of arterial stiffness gradient has not been thoroughly studied.

Therefore, we aimed to examine the effect of acute administration of NTG on arterial stiffness gradient. Second, we aimed to study the effect of NTG on local stiffness parameters of the brachial artery and its relation to changes in cr-PWV. Finally, we investigated whether the vascular response to NTG is different in patients with CKD than subjects without a significant decline in renal function.

## Materials and Methods

### Study Design and Population

This is an interventional study designed to examine the acute effects of NTG in a single session of hemodynamic assessment. The inclusion criteria were adult patients with stable blood pressure (BP) medication and without any acute episode of illness (infection or recent cardiovascular events). Patients were excluded if they had a history of adverse reactions to NTG or were on renal replacement therapy. The control group (*n* = 55) was composed of apparently healthy subjects or hypertensive subjects with an estimated glomerular filtration rate (eGFR) ≥ 45 ml/min/1.73 m^2^ [CKD-EPI equation (Levey et al., 2009)]. The CKD group (*n* = 38) was composed of patients with an eGFR < 45 ml/min/1.73 m^2^ (i.e., Stage 3B CKD and above) (Kidney Disease Improving Global Outcomes [KDIGO], 2013). This cutoff was used because subjects with an eGFR ≥ 45 ml/min/1.73 m^2^ are not at substantially higher risk of cardiovascular disease (Padhy et al., 2017). The participants recruited were healthcare workers and patients treated in hypertension or CKD clinics of CHU de Québec-Université Laval hospital. Hypertension was defined as BP ≥ 140/90 mmHg or the use of antihypertensive medications in patients with a history of hypertension. Cardiovascular disease was defined as myocardial infarction, coronary artery revascularization, or ischemic heart disease as shown by treadmill, echo, or thallium stress tests; stroke or lower extremity amputation; or revascularization. This protocol had been approved by the *comité d’éthique de la recherche du CHU de Québec* and was conducted in accordance with the Declaration of Helsinki. All procedures followed were in accordance with institutional guidelines, and each patient had provided informed written consent.

### Hemodynamic Measurements Sequence

All measurements were performed after 15 min of rest in a supine position. Brachial artery BP was recorded using an automatic oscillometric sphygmomanometer BPM-100 (BP-Tru, Coquitlam, Canada) by an experienced operator who was present in the room. BP was recorded six times, with a 2-min interval between each measurement, and the average of the last five measurements was used to determine the brachial systolic BP (SBP), diastolic BP (DBP), and brachial artery pulse pressure (*b*PP). Immediately after BP measurements, radial pulse wave profile was recorded using applanation tonometry after calibration with brachial SBP and DBP (SphygmoCor system ^®^, AtCor Medical Pty. Ltd., Sydney, Australia), followed by the determination of PWVs in peripheral (brachial and femoral) and central (aortic) arterial segments using Complior ^®^ Analyse (Artech Medical, Pantin, France). Brachial BP, PWVs, and radial pulse wave profile measurements were repeated 5 min after the sublingual administration of 0.4 mg of NTG with the same described procedures except that pulse wave profile has been taken once instead of thrice. Radial pulse wave profile post-NTG was introduced later in the measurement protocol and performed in the last 56 patients. Wall properties of the brachial artery were assessed in 57 subjects by a radiofrequency-based wall-tracking system (ART-LAB, Esaote, Maastricht, Netherlands) before and continuously during 10 min after NTG administration.

### Pulse Wave Velocity and Arterial Stiffness Gradients

We determined PWVs by direct measurements of distance and using a maximal upstroke algorithm for pulse transit time. Carotid-radial PWV (cr-PWV, carotid-dorsalis pedis PWV (cp-PWV), and carotid-femoral PWV (cf-PWV were obtained on the same side, in triplicates. Femoral-dorsalis pedis PWV was calculated using the formula (cp distance − cf distance)/(cp transit time − cf transit time). Stiffness gradients from the aorta to the brachial artery (upper limb) and from the aorta to the femoral artery (lower limb) were obtained using the aortic-brachial PWV ratio (AB-PWV ratio = cf-PWV/cr-PWV) and aortic-femoral PWV ratio (AF-PWV ratio = cf-PWV/fp-PWV), respectively.

### Local Brachial Stiffness Parameters

With the right arm stabilized in an inflated cushion, pre-NTG end-diastolic diameter (*d*), intima-media thickness (IMT), and distension of the brachial artery (∼3 cm above the elbow crease). The ART-LAB probe (10 MHz linear probe) was fastened by a locking articulating arm mounted on a platform that allowed two-dimensional microadjustments and good stability for a 10 min-period of real-time measurements after sublingual NTG administration. Arterial wall properties were calculated as follows:


(1) $$\;•\text{Compliance}=\frac{△\,A}{△\,P}=\frac{A_{s}-A_{d}}{b\,P\,P},\text{where}\,A_{s}\,\text{and}\,A_{d}\,\mathrm{stand}\,\mathrm{for}\,\mathrm{the}\,\mathrm{systolic}\,\mathrm{and}\,\mathrm{diastolic}\,\mathrm{cross}\,\text{-}\,\mathrm{sectional}\,\mathrm{areas};$$

(2) $$\;•\mathrm{Distensibility}\,\left(\mathrm{DC}\right)=\frac{1}{A_{d}}\cdot \frac{△\,A}{△\,P};$$

(3) $$\;•\mathrm{Luminal}\,\mathrm{cross}\,\text{-}\,\mathrm{sectional}\,\mathrm{area}\,\left(\mathrm{LCSA}\right)=\frac{\pi \,d^{2}}{4};$$

(4) $$\;•\mathrm{Mean}\,\mathrm{wall}\,\mathrm{cross}\,\text{-}\,\mathrm{sectional}\,\mathrm{area}\,\left(\mathrm{WCSA}\right)=\pi \,{\left(\frac{d+2\times I\,M\,T}{2}\right)}^{2}-\pi \,{\left(\frac{d}{2}\right)}^{2};$$

(5) $$\;•\mathrm{Incremental}\,\mathrm{elastic}\,\mathrm{modulus}\,\left({\text{E}}_{\text{inc}}\right)=\frac{3}{D\,C}\,\left(1+\frac{L\,C\,S\,A}{W\,C\,S\,A}\right);$$

(6) $$\;•\mathrm{Wall}\,\mathrm{to}\,\mathrm{lumen}\,\mathrm{ratio}\,\left(W/\mathrm{Lratio}\right)=2\,\left(\frac{I\,M\,T}{d}\right);$$

(Laurent et al., 1994, 2006; Bortolotto et al., 1999; Spronck et al., 2021).

The IMT measurements were not performed after NTG administration, but it was calculated based on the principle of WCSA conservation.

### Central Pulse Wave Profile Analysis

Three consecutive recordings were performed on the radial artery to construct a central pulse wave profile using an integrated generalized transfer function. The average of central SBP, central DBP, central pulse pressure (PP), heart rate-adjusted central augmented pressure (AP@75), and augmentation index (AIx@75), as well as end systolic pressure, were measured. Period, ejection duration, diastolic duration, the first peak of pressure height (P1) with the respective time of each peak (T1, T2), time to the reflection of the reflected pressure wave (TR), and subendocardial viability ratio (SEVR) were also obtained (Miyashita, 2012).

### Statistical Analysis

The results are expressed as mean ± SD, *n* (%), or median (25th–75th percentiles). Differences between groups were assessed using *t*-test, Mann-Whitney *U*-test, or chi-square accordingly. The effects of NTG on vascular parameters were examined using paired *t*-test. Linear regression was used to examine the relationship between renal function, PWVs, and PWV ratios. We used generalized estimating equations (GEE) to adjust changes in vascular parameters for changes in DBP. Linear regressions were performed to determine the relationship between cr-PWV and brachial artery diameter. GEEs were also used to compare if the vascular response to NTG was different between CKD and the control group. Forward conditional linear regression was used to identify the determinants of changes in cr-PWV after NTG administration. We used the Spearman rank correlation coefficient to assess the association between TR and AP@75 with both AB-PWV and AF-PWV ratios. Finally, as part of sensitivity analysis, we examined if there were any significant interactions between age, sex, and established cardiovascular disease, and the vascular response to NTG (GEE). All statistical analyses were performed using SPSS Statistics for Windows, Version 27.0 (IBM Corp., Armonk, NY, United States). A two-tailed *p*-value <0.05 was considered to be statistically significant.

## Results

### Patient Population

Table 1 shows the baseline demographic, clinical, pharmacological, and biological characteristics of patients overall and in control and CKD subjects, respectively. Patients with CKD were older; had a higher BMI and triglyceride levels; had a higher prevalence of hypertension, cardiovascular disease; and diabetes; and were more frequently treated using calcium-channel blockers and statins. At baseline, there were significant correlations between eGFR, cf-PWV (*r* = − 0.37, *p* < 0.001), AB-PWV ratio (*r* = − 0.38, *p* < 0.001), AF-PWV ratio (*r* = − 0.27, *p* = 0.024), but not with cr-PWV (*r* = − 0.03, *p* = 0.76), and fp-PWV (*r* = − 0.12, *p* = 0.33) in this study population.

**TABLE 1:** Characteristics of patients for control group, CKD group, and overall.

	Overall	Control	CKD
		eGFR ≥ 45 ml/min/1.73 m^2^	eGFR < 45 ml/min/1.73 m^2^
Parameters	*n* = 93	*n* = 55	*n* = 38
Male	62 (67)	35 (64)	27 (71)
Age (y)	61.0 [42.8–74.1]	57.1 [39.0–68.0]	72.0 [60.1–79.0]*
Weight (kg)	77.5 ± 15.0	76.2 ± 16.2	79.3 ± 13.0
Body mass index (kg/m^2^)	27.1 ± 4.3	26.3 ± 4.5	28.3 ± 3.8*
Hypertension	60 (65)	26 (47)	34 (90)*
Diabetes	15 (16)	5 (9)	10 (26)*
CVD	12 (13)	3 (6)	9 (24)*
eGFR (ml/min/1.73 m^2^)	43.0 [21.3–80.0]	80.0 [64.5–96.0]	22.0 [10.0–35.3]*
**Medication**			
ACEi/ARB	46 (50)	25 (46)	21 (55)
CCB	34 (37)	13 (24)	21 (55)*
β-blockers	20 (21)	10 (18)	10 (26)
ASA	22 (24)	13 (24)	9 (24)
Statins	38 (41)	14 (26)	24 (63)*
**Lipid profile**			
Total cholesterol (mmol/L)	4.61 ± 1.00	4.43 ± 0.86	4.79 ± 1.09
HDL (mmol/L)	1.37 ± 0.39	1.40 ± 0.32	1.34 ± 0.45
LDL (mmol/L)	2.30 ± 0.80	2.29 ± 0.84	2.30 ± 0.76
TG (mmol/L)	2.02 ± 1.16	1.62 ± 0.69	2.44 ± 1.38*

*Results are means ± SD, n (%) or median (25th–75th percentiles). The p-value <0.05 is statistically significant.*

*ACEi, angiotensin-converting enzyme inhibitor; ARB, angiotensin receptor blockers; ASA, acetylsalicylic acid; CCB, calcium-channel blockers; CVD, cardiovascular disease; eGFR, estimated glomerular filtration rate from CKD-EPI equations; HDL, high-density lipoprotein; LDL, low-density lipoprotein; TG, triglycerides.*

*A p-value of <0.05 than control group.

As shown in Table 2, after NTG administration, there was a slight, but statistically significant, decline in SBP, DBP, and PP. In the 56 subjects who had central pulse wave analysis before and after NTG administration, there was a reduction in AP@75 (8.8 ± 7.4 vs. 3.6 ± 5.6 mmHg; *p* < 0.001) and AIx@75 (19.7 ± 13.7 vs. 7.4 ± 14.8%; *p* < 0.001). The end-systolic pressure decreased despite a reduction in ejection duration, but no changes were observed in the period. Finally, there was an increase in SEVR (156.6 ± 29.0 vs. 163.4 ± 31.3%; *p* = 0.004).

**TABLE 2:** Nitroglycerin-induced changes in brachial pressures and regional arterial stiffness.

	Baseline *n* = 93	NTG *n* = 93	*p*-value	*p*-value (adjusted for DBP)
**Peripheral pressure parameters**				
SBP (mm Hg)	123.8 ± 18.8	119.2 ± 17.2	<0.001	
DBP (mm Hg)	73.1 ± 9.2	71.9 ± 10.0	0.028	
PP (mm Hg)	50.7 ± 16.1	47.3 ± 13.0	<0.001	
MBP (mm Hg)	92.2 ± 11.5	89.4 ± 12.2	<0.001	
Heart rate (bpm)	65.3 ± 11.1	66.2 ± 10.6	0.025	
**Central pressure parameters (*n* = 56)**				
SBP (mm Hg)	115.3 ± 20.8	108.3 ± 16.6	<0.001	
DBP (mm Hg)	74.8 ± 8.8	73.5 ± 9.0	0.065	
PP (mm Hg)	41.7 ± 18.4	34.8 ± 14.2	<0.001	
AP@75 (mm Hg)	8.8 ± 7.4	3.6 ± 5.6	<0.001	
Aix@75 (%)	19.7 ± 13.7	7.4 ± 14.8	<0.001	
P1 (mmHg)	29.2 ± 9.1	28.1 ± 7.6	0.160	
T1 (ms)	105.0 ± 9.5	109.9 ± 13.9	0.009	
TR (ms)	139.8 ± 12.1	146.1 ± 14.9	<0.001	
T2 (ms)	228.0 ± 24.7	211.2 ± 27.7	<0.001	
End-systolic pressure (mm Hg)	104.1 ± 16.7	95.8 ± 15.3	<0.001	
Ejection duration (ms)	279.7 ± 59.2	267.5 ± 67.0	<0.001	
Diastolic duration (ms)	634.7 ± 133.9	644.1 ± 137.0	0.228	
Period (ms)	965.1 ± 156.3	968.2 ± 155.9	0.696	
SEVR (%)	156.6 ± 29.0	163.4 ± 31.3	0.004	
PP amplification	1.33 ± 0.20	1.47 ± 0.22	<0.001	
**Pulse wave velocities**				
cf-PWV (m/s)	12.12 ± 3.36	12.14 ± 3.55	0.928	0.236
cr-PWV (m/s)	9.51 ± 1.81	8.65 ± 1.57	<0.001	<0.001
cp-PWV (m/s)	9.97 ± 1.67	9.02 ± 1.24	<0.001	<0.001
fp-PWV (m/s)	9.71 ± 1.89	8.14 ± 1.62	<0.001	0.032
Aortic-brachial PWV ratio	1.30 ± 0.39	1.42 ± 0.46	0.001	0.001
Aortic-femoral PWV ratio	1.38 ± 0.47	1.56 ± 0.53	0.001	0.005

*Results are means ± SD. The p-values were obtained with paired-samples t-test and MBP-adjusted PWVs were obtained with generalized estimating equations.*

*The p-value < 0.05 is statistically significant.*

*Aix@75, heart rate-adjusted augmentation index; AP@75, heart rate-adjusted augmented pressure; cp-PWV, carotid-dorsalis pedis pulse wave velocity; cr-PWV, carotid-radial pulse wave velocity; DBP, diastolic blood pressure; fp-PWV, femoral-dorsalis pedis pulse wave velocity; MBP, mean blood pressure; NTG, nitroglycerin; P1, first peak of pressure height; PP, pulse pressure; PP amplification, pulse pressure amplification; SBP, systolic blood pressure; SEVR, subendocardial viability ratio; T1, time of peak 1; T2, time of peak 2; TR, time to reflection of the reflected pressure wave.*

### Impact of Nitroglycerin on Regional Stiffness and Arterial Stiffness Gradients

**FIGURE 1:**
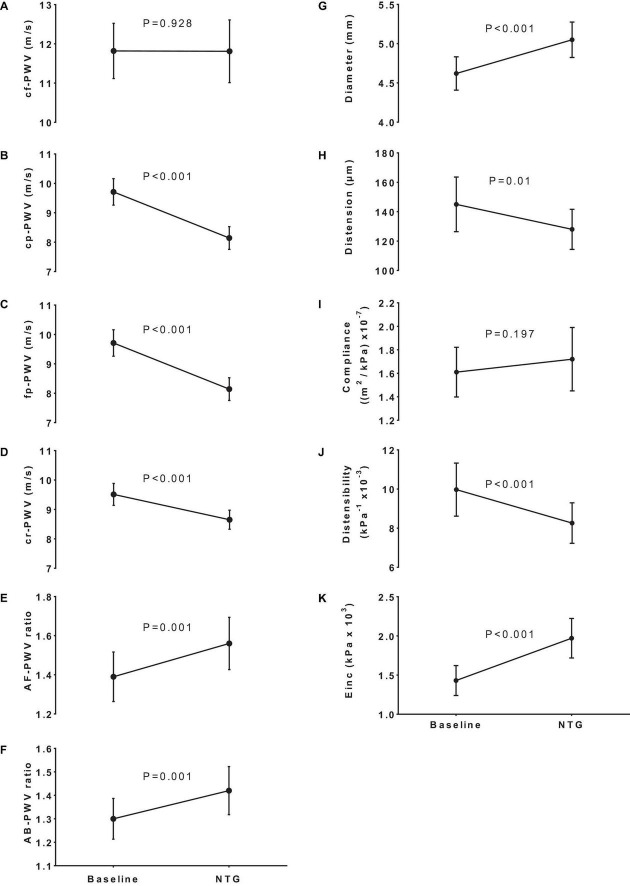
Impact of NTG on regional stiffness, arterial stiffness gradients, and local brachial stiffness parameters. Panels **(A–D)** show the effect of nitroglycerin (NTG) on pulse wave velocity of various territories (PWV). The NTG had no effect on carotid-femoral PWV (cf-PWV) but significantly decreased carotid-pedis PWV (cp-PWV), femoral-pedis PWV (fp-PWV), and carotid-radial PWV (cr-PWV) leading to a corresponding increase in aortic-femoral PWV ratio [AF-PWV ratio **(E)**] and aortic-brachial PWV ratio [AB-PWV ratio **(F)**]. Changes in the brachial artery diameter **(G)**, distention **(H)**, compliance **(I)**, distensibility **(J)**, and incremental elastic modulus [E_inc_,**(K)**] in response to NTG.

As shown in Table 2 and Figures 1A–D, after NTG administration, there was no significant change in cf-PWV (*p* = 0.928), but there were significant reductions in both cr-PWV (0.86 ± 1.27 m/s; *p* < 0.001) and fp-PWV (1.12 ± 1.74 m/s; *p* < 0.001). The results remained consistent even after adjustment for the small changes in DBP (Table 2). Consequently, as shown in Figures 1E,F, both AB-PWV and AF-PWV ratios increased significantly after NTG administration and remained significant even after adjustment for changes in DBP.

Table 3 shows the correlation matrix between changes in AB-PWV and AF-PWV ratios and the TR and AP@75.

**TABLE 3:** Correlations between changes in arterial stiffness gradient and changes in wave reflection parameters.

	Δ AB-PWV ratio		Δ AF-PWV ratio	
	*r*	*p*-value	*r*	*p*-value
Δ TR	0.203	0.140	0.289	0.042
Δ AP@ 75	−0.288	0.032	−0.467	<0.001

*Correlation and p-values were obtained using Spearman rank bivariate correlation. The p-value <0.05 is statistically significant.*

*AB-PWV, aortic-brachial pulse wave velocity; AF-PWV, aortic-femoral pulse wave velocity; TR, time to the reflection of the reflected pressure wave.*

### Local Brachial Stiffness in Response to Nitroglycerin

Table 4 describes the changes in the brachial artery diameter and stiffness parameters in response to NTG. As expected, after NTG, the brachial artery diameter (4.62 ± 0.80 vs. 5.18 ± 0.77 mm; *p* < 0.001) and the luminal cross-sectional area (17.2 ± 5.9 vs. 21.6 ± 6.2 mm^2^; *p* < 0.001) increased significantly. However, systolic-diastolic distention (0.145 ± 0.064 vs. 0.128 ± 0.047 mm; *p* = 0.010) and distensibility (9.97 ± 4.67 vs. 8.26 ± 3.56 kPa^–1^ × 10^–3^; *p* < 0.001) decreased, incremental elastic modulus increased (1.43 ± 0.65 vs. 1.97 ± 0.86 kPa^–1^ × 10^3^; *p* < 0.001), without any significant changes in brachial artery compliance (1.61 ± 0.72 vs. 1.72 ± 0.92 m^2^⋅kPa^–1^ × 10^–7^; *p* = 0.197). These results are summarized in Figures 1G–K.

**TABLE 4:** Brachial artery changes according to nitroglycerin administration.

	Baseline *n* = 57	NTG *n* = 57	*p*-value	Δ NTG-baseline	*p*-value (adjusted for DBP)
**Brachial artery**					
Diameter (mm)	4.62 ± 0.80	5.18 ± 0.77	<0.001	0.57 (0.50 to 0.64)	<0.001
IMT (mm)	0.385 ± 0.076	0.346 ± 0.072*	–	−0.037 (−0.42 to −0.33)	–
W/L ratio	0.17 ± 0.03	0.14 ± 0.03	<0.001	−0.03 (−0.03 to −0.02)	<0.001
LCSA (mm^2^)	17.2 ± 5.9	21.6 ± 6.2	<0.001	4.33 (3.80 to 4.86)	<0.001
WCSA (mm^2^)	6.13 ± 1.92	–	–	–	–
Distension (mm)	0.145 ± 0.064	0.128 ± 0.047	0.010	−0.017 (−0.029 to −0.004)	0.012
Compliance (m^2^⋅ kPa^–1^ × 10^–7^)	1.61 ± 0.72	1.72 ± 0.92	0.197	0.11 (−0.06 to 0.29)	0.254
Distensibility (kPa^–1^ × 10^–3^)	9.97 ± 4.67	8.26 ± 3.56	<0.001	−1.72 (−2.69 to −0.75)	0.006
Einc (kPa × 10^3^)	1.43 ± 0.65	1.97 ± 0.86	<0.001	0.55 (0.37 to 0.72)	<0.001

*Results are means ± SD or (95% CI). The p-values and mean changes in parameters with 95% CI were obtained using the paired-samples t-test, and MBP-adjusted p values were obtained using generalized estimating equations. The p-value <0.05 is statistically significant.*

E_inc_, incremental elastic modulus; WCSA, mean wall cross-sectional area; IMT, intima media thickness; LCSA, luminal cross-sectional area; W/L ratio, wall-to-lumen ratio. *Post-NTG IMT was not measured but calculated based on the conservation of wall cross-sectional area.

### Brachial Pulse Wave Velocity and Brachial Diameter

To examine if the change in cr-PWV was related to brachial artery diameter, we performed a linear regression analysis and found that higher baseline cr-PWV and smaller baseline diameter were significant determinants in the decline of cr-PWV after NTG administration (*R*^2^ = 0.42; Table 5). Age, CKD, diabetes, and cardiovascular disease were not associated with changes in cr-PWV after NTG. We also examined and found no direct relationship between changes in cr-PWV and changes in brachial diameter or wall-to-lumen ratio in either absolute or relative terms.

**TABLE 5:** Determinants of change in carotid-radial PWV after NTG.

Parameters	Slope (95% Confidence interval)	*p*-value	*R* ^2^
**Final model**			0.421
Constant	−0.115 (−2.315 to 2.085)	0.917	
Baseline cr-PWV (m/s)	−0.426 (−0.614 to −0.237)	<0.001	
Baseline brachial diameter (mm)	0.708 (0.371 to 1.046)	<0.001	

*The final model of a forward conditional regression analysis where excluded variables (e.g., age, CKD, diabetes, and cardiovascular status) are not shown. The p-value <0.05 is statistically significant.*

### Vascular Response to Nitroglycerin by Chronic Kidney Disease Status

Given the clinical heterogeneity of subjects with or without reduction in renal function, we examined if the vascular response to NTG could be affected by the assigned group. To examine this, we built an interaction term into the GEE equations and found no significant interaction between CKD status and changes in PWVs, PWV ratios, and brachial parameters after NTG.

In addition, we examined and found no modifying effect of age (median 61 years old), sex, and established cardiovascular disease on the extent of studied vascular responses to NTG.

## Discussion

This study shows that acute administration of NTG reduces brachial and femoral PWVs without affecting aortic PWV, causing an increase in the AB-PWV and AF-PWV ratios. The delay in the timing of the reflected wave and the lowering of the amplitude of the reflection wave after NTG were best correlated with the changes in the AF-PWV ratio.

These observations are in line with previous studies showing that NTG reduces PWV in muscular peripheral arteries but has no effect in attenuating aortic PWV (Pauca et al., 2005; Yamamoto et al., 2017). These changes result in the attenuation or even the reversal of the arterial stiffness gradient. From a cardiac point of view, increasing the AB-PWV and AF-PWV ratios and delaying the arrival and amplitude of the reflection wave in the ascending aorta may seem desirable, especially in the context of heart failure where nitrates and direct vasodilator (hydralazine) have been shown to improve left ventricular ejection fraction, peak oxygen consumption, and mortality (Cohn et al., 1991; Taylor et al., 2004). However, based on the wave propagation model, if there is less wave reflection in the ascending aorta, the forward pressure wave is less attenuated along the arterial tree, leading to a higher pulsatile pressure transmitted to the microcirculation. As stated earlier, given that the microcirculation is not structurally designed to accommodate a high pulse pressure, its chronic exposure to such mechanical stress may lead to microvascular dysfunction and end-organ damage. This is especially problematic for high flow/low resistance organs such as the brain and the kidneys (Mitchell, 2008). Indeed, in type 2 diabetic patients, Pierce et al. (2016) have observed that aortic-brachial stiffness gradient was associated with kidney function independent of aortic stiffness, implying that the arterial stiffness gradient may have a pathophysiological role in kidney disease. In addition, Mitchell et al. (2011) have shown that the loss of aortic-carotid impedance mismatch reduced the carotid reflection coefficient and facilitated the transmission of excessive flow pulsatility into the brain vasculature, which is presumably responsible for structural injuries to the brain microvasculature and the ensuing cognitive decline.

The clinical and biological determinants of a lower cr-PWV remain largely unknown and understudied. However, data are emerging that in certain conditions such as heart failure, kidney disease, or obesity in adolescents, the cr-PWV may be lower, hence contributing to the attenuation of the arterial stiffness gradient beyond the sole increase in aortic PWV (Mitchell et al., 2001; Zhu et al., 2007; Pierce et al., 2013, 2016; Utescu et al., 2013; Fortier et al., 2015; Agbaje et al., 2021). Moreover, the subjects of the African Americans of the atherosclerosis risk in communities (ARIC) study, tended to have a less favorable arterial stiffness gradient, not only due to higher cf-PWV but also because they presented with lower femoral-ankle PWV than Caucasians (Meyer et al., 2016).

These findings may suggest that the use of surrogate markers of arterial stiffness gradient may have a superior prognostic value above and beyond aortic stiffness. For example, in the dialysis population, we and others have shown that a higher AB-PWV ratio is associated with the worst survival rate even after adjustments for potential confounding factors (Fortier et al., 2015; Bao et al., 2019). In addition, the AB-PWV ratio was associated with cardiovascular events after kidney transplantation (*p* = 0.045) but not cf-PWV (*p* = 0.501), and only the AB-PWV ratio has been associated with eGFR and albuminuria in a cohort of middle-aged women with increased cardiovascular risk (Laucyte-Cibulskiene et al., 2018, 2019). However, the superiority of the AB-PWV ratio over cf-PWV was not observed in patients at earlier stages of kidney disease (Bai et al., 2019). It is suggested that this is likely because the AB-PWV ratio increases significantly in patients once the eGFR decreases below 45 ml/min/1.73 m^2^ (i.e., Stage 3B and above) (Zanoli et al., 2018).

In the ARIC study population, a cross-sectional analysis showed that a low arterial stiffness gradient (femoral-ankle PWV/cf-PWV) was associated with diabetes, coronary heart disease, heart failure, and stroke, whereas a high cf-PWV was only associated with hypertension (Stone et al., 2021). It should be mentioned that this is a relatively older cohort (age of 75 years) with a higher prevalence of hypertension (72%) and diabetes (29%). In contrast, the AB-PWV ratio was not superior to cf-PWV in predicting cardiovascular events in subjects from the Framingham Heart Study, a younger (mean age of 60 years) and healthier cohort (8% diabetes, 31% receiving antihypertensive drugs) (Niiranen et al., 2017).

In this study, NTG resulted in vasodilation of the brachial artery (12%), but this increase in diameter was accompanied by a lower degree of distention, showing no significant changes in the brachial artery compliance and even an increase in incremental elastic modulus. This increase in incremental elastic modulus could be explained by the transfer of pressure load from the vascular smooth muscle cells to the stiffer collagens of the extracellular matrix (Roach and Burton, 1957). Therefore, the reduction in cr-PWV could have potentially been explained by a decrease in a wall-to-lumen ratio according to the Moens–Korteweg equation $P\,W\,V=\sqrt{\frac{E\,i\,n\,c\cdot h}{2\,r\cdot \rho }}$, where *h* is the wall thickness and *r* is the radius of the artery, and ρ is the blood density. However, we found no statistical relationship between measured changes in cr-PWV and changes in either diameter or wall-to-lumen ratio. This discrepancy could be explained by several reasons. First, it could be that our spatial resolution was too limited for a reliable assessment of *in vivo* changes in wall-to-lumen ratio. Second, it could be related to the violation of basic assumptions underlying the equation, i.e., that the arterial wall is isotropic and behaves in an isovolumetric manner in response to pulse pressure (Gosling and Budge, 2003). In addition, since *in vivo* arterial diameter at zero transmural pressure could not be measured, Hooke’s law was applied for the determination of E_inc_ (Roark and Young, 1989), therefore implying geometrical assumptions that the arterial segments were considered as uniform thick-walled tubes. However, this assumption does not take into account any geometrical variation along the arterial wall. Finally, this discrepancy can be explained by the heterogeneity of the arterial tree between carotid and radial artery both in terms of diameter, arterial wall thickness, and composition. As such, the measured PWV over an arterial path is the summation of the local vascular responses along with the studied segments (i.e., ignoring potential heterogeneity of the vascular response). This possibility is further supported by the results from the PWV between the carotid and dorsal pedis artery, which declined after NTG, but this reduction was solely due to the reduction over the vascular bed below the femoral artery.

Besides arterial stiffness gradient, this study underlines the importance of proper assessment of regional vascular stiffness and its response to therapy. Given the nature of translational medicine, we have been eager to take the arterial stiffness assessments out of specialized laboratories and into the large-scale epidemiological studies and clinical practice through simplification of arterial stiffness assessment. For example, our study clearly shows that PWV from the carotid to the dorsal pedis artery declined significantly after NTG, but upon closer look, we saw that this decline was related to the reduction in PWV from the femoral to the dorsal pedis segment and not related to aortic PWV *per se*. This observation is in line with the findings of Yamamoto et al. (2017) using cardio-ankle vascular index (CAVI) and heart-thigh and thigh-ankle B-stiffness indices.

This study has several strengths and limitations. First, it involves a large number of subjects with a detailed evaluation of various regional pulse wave velocities, central pulse wave profile, and brachial stiffness parameters. Second, given the heterogeneity of the subjects, we were able to perform various sensitivity analyses showing the robustness of our findings. Indeed, older subjects may have a more hypotensive response to NTG (Cahalan et al., 1992). However, even if our CKD patients were older and had several other heterogeneities, it did not modify the vascular responses in this study. There are also limitations that should be mentioned. First, we did not examine the smaller arteries of the vascular bed through the assessment of radial-digital PWV (Obeid et al., 2021). Second, it would have also been interesting to assess the local distensibility of both carotid and femoral arteries. However, given that we only had a 5-min window to measure all PWVs in triplicates and brachial distensibility, the addition of local carotid and femoral distensibilities would have pushed us outside the desired window, and besides technical limitations, it would have added uncertainties regarding the potential fading effect of NTG. Third, our interpretation of results is based on the pulse wave propagation model, which does not consider the reservoir function of the arterial tree (Wang et al., 2003; Tyberg et al., 2009; Parker et al., 2012; Mynard and Smolich, 2014). In addition, there are two limitations regarding the use of augmentation index (AIx) as a marker of wave reflection, namely, the reliability of the central AIx estimated *via* transfer function from the radial pressure waveform and the validity of central AIx as a measure of wave reflection (Chen et al., 1996; Millasseau et al., 2003; Segers et al., 2005; Miyashita, 2012; Hughes et al., 2013; Heusinkveld et al., 2019). Indeed, the central AIx derived from the generalized transfer function seems to correlate more strongly with the AIx of the original peripheral waveform rather than the central pressure waveform (Segers et al., 2005). Furthermore, the AIx and the related arrival time may be influenced by other factors, such as myocardial contractility, and hence may not be a proper measure of wave reflection (Hughes et al., 2013; Heusinkveld et al., 2019). Finally, our results are limited to the vascular response to NTG in an acute setting and do not imply similar effects would necessarily occur in a chronic setting.

## Conclusion

While there are statistical associations between markers of arterial stiffness gradient and certain clinical conditions in both cross-sectional and longitudinal studies, there has not been a systematic focus on the impact of pharmacological interventions on the arterial stiffness gradient. In light of recent evidence supporting the potential usefulness of arterial stiffness gradient assessment, and our present data showing an alteration in arterial stiffness gradient in response to NTG, we would like to underline the importance of assessing regional PWV in order to develop an integrated approach into the arterial tree and its response to therapy. As such, these results are hypothesis generating and their clinical and pharmacological usefulness should be assessed in properly designed studies.

## Data Availability Statement

The raw data supporting the conclusions of this article will be made available by the authors, without undue reservation.

## Ethics Statement

The studies involving human participants were reviewed and approved by Comité d’Éthique de la Recherche du CHU de Québec. The patients/participants provided their written informed consent to participate in this study.

## Author Contributions

MA and CF provided the conception and design of the study. CF, C-AG, MP, HO, NC, KD, and MA recruited participants and collected data. CF, C-AG, and MA performed the statistical analysis and wrote the manuscript. All authors contributed to manuscript revision, read, and approved the submitted version.

## Conflict of Interest

The authors declare that the research was conducted in the absence of any commercial or financial relationships that could be construed as a potential conflict of interest.

## Publisher’s Note

All claims expressed in this article are solely those of the authors and do not necessarily represent those of their affiliated organizations, or those of the publisher, the editors and the reviewers. Any product that may be evaluated in this article, or claim that may be made by its manufacturer, is not guaranteed or endorsed by the publisher.
